# Comparative Analysis of the Microbiota Between Sheep Rumen and Rabbit Cecum Provides New Insight Into Their Differential Methane Production

**DOI:** 10.3389/fmicb.2018.00575

**Published:** 2018-03-27

**Authors:** Lan Mi, Bin Yang, Xialu Hu, Yang Luo, Jianxin Liu, Zhongtang Yu, Jiakun Wang

**Affiliations:** ^1^Laboratory of Ruminant Nutrition, Institute of Dairy Science, College of Animal Sciences, Zhejiang University, Hangzhou, China; ^2^Department of Animal Sciences, The Ohio State University, Columbus, OH, United States

**Keywords:** acetogen, cecum, fibrolytic bacteria, hydrogen, methane, microbiota, pH, rumen

## Abstract

The rumen and the hindgut represent two different fermentation organs in herbivorous mammals, with the former producing much more methane than the latter. The objective of this study was to elucidate the microbial underpinning of such differential methane outputs between these two digestive organs. Methane production was measured from 5 adult sheep and 15 adult rabbits, both of which were placed in open-circuit respiratory chambers and fed the same diet (alfalfa hay). The sheep produced more methane than the rabbits per unit of metabolic body weight, digestible neutral detergent fiber, and acid detergent fiber. pH in the sheep rumen was more than 1 unit higher than that in the rabbit cecum. The acetate to propionate ratio in the rabbit cecum was more than threefold greater than that in the sheep rumen. Comparative analysis of 16S rRNA gene amplicon libraries revealed distinct microbiota between the rumen of sheep and the cecum of rabbits. Hydrogen-producing fibrolytic bacteria, especially *Butyrivibrio*, *Succiniclastium*, *Mogibacterium*, *Prevotella*, and *Christensenellaceae*, were more predominant in the sheep rumen, whereas non-hydrogen producing fibrolytic bacteria, such as *Bacteroides*, were more predominant in the rabbit cecum. The rabbit cecum had a greater predominance of acetogens, such as those in the genus *Blautia*, order *Clostridiales*, and family *Ruminococcaceae*. The differences in the occurrence of hydrogen-metabolizing bacteria probably explain much of the differential methane outputs from the rumen and the cecum. Future research using metatranscriptomics and metabolomics shall help confirm this premise and understand the factors that shape the differential microbiota between the two digestive organs. Furthermore, our present study strongly suggests the presence of new fibrolytic bacteria in the rabbit cecum, which may explain the stronger fibrolytic activities therein.

## Introduction

Mammalian herbivores do not synthesize the enzymes needed to digest cellulose or hemicellulose. They depend on a symbiotic relationship with a community of microbes (primarily bacteria) with fibrolytic ability in either their foregut (i.e., the rumen of ruminants and the pseudo-ruminants) or their hindgut (i.e., the cecum and colon of non-ruminant herbivores) for fiber digestion (Furness et al., 2015). Both foregut and hindgut fermenters produce methane (CH_4_) as an inevitable by-product during feed fermentation. As a greenhouse gas, CH_4_ is 23 times more potent than carbon dioxide (CO_2_) (IPCC, 2014). A significant portion of the ingested feed energy is also lost as CH_4_, ranging from 1.5 to 12% of the gross energy intake in cattle (Johnson and Johnson, 1995; Franz et al., 2010). Ruminants are the main producing animals of meat and milk, but they also produce more CH_4_ than monogastric animals per unit of BW^0.75^ or product (Franz et al., 2010). Indeed, up to 20% of the global anthropogenic CH_4_ is emitted by ruminants (Bhatta et al., 2007). Intensive research has aimed to mitigate CH_4_ emission to ensure sustainable production of beef, lamb, and dairy products.

Methanogenesis in the rumen and hindgut is predominately driven via the hydrogenotrophic pathway using hydrogen (H_2_) and CO_2_ (also formate) as the substrates (Liu and Whitman, 2008) though some CH_4_ is also produced through the methylotrophic methanogenesis pathway using methanol and methylamines as the substrates (Poulsen et al., 2013). The genus *Methanobrevibacter* is the most ubiquitous and predominant hydrogenotrophic methanogens found in the foregut and hindgut of herbivores (Kušar and Avguštin, 2010; St-Pierre and Wright, 2013) although several species of *Methanomassiliicoccales* can use methyl substrates (Poulsen et al., 2013). Although affected by several factors, such as pH, the rate and CH_4_ output from herbivores are primarily determined by the availability of methanogenic substrates (i.e., H_2_ and CO_2_), which are in turn determined by the rates of production and consumption. Fermentative acetate production accompanied with H_2_ production is thermodynamically favored, especially when forage-based diets are fed because more ATP is synthesized (Russell and Rychlik, 2001). CH_4_ output can vary among cows or sheep fed the same diet (Kittelmann et al., 2014; Wallace et al., 2015), and CH_4_ output was found to be positively associated with bacterial populations that ferment ingested feed to relatively more hydrogen in sheep (Kittelmann et al., 2014). Furthermore, hydrogenotrophic methanogens are thermodynamic favored than acetogens when competing for hydrogen in rumen (Cord-Ruwisch et al., 1988; Joblin, 1999), but in the foregut (tubular) of kangaroos, acetogens outcompete methanogens for CO_2_ and H_2_ and can synthesize acetate via the acetyl-CoA pathway, providing a significant energetic benefit to the host animal (Attwood and McSweeney, 2008). Similar hydrogen disposal pathway was thought to be present in the cecum of rabbits, but no acetogens were reported (Piattoni et al., 1996). In a recent study, species of *Blautia* including *B. coccoides*, *B. hydrogenotrophica*, and *B. schinikii*, which are known acetogens, were found at high predominance in rabbit cecum (Yang et al., 2016). We hypothesized that the hindgut of hindgut fermenters probably also has a distinct microbiota than the rumen of ruminants, and such difference may be the main reason for the differential CH_4_ outputs between these two types of herbivores. In the present study, we tested this hypothesis using sheep as ruminants and New Zealand White (NZW) rabbits as a non-ruminant herbivore, with alfalfa hay as the only diet. Feed consumption, fermentation characteristics, CH_4_ emission, and the microbiota in the sheep rumen and the rabbit cecum were comparatively analyzed. The differences determined in the above measurements will help understand the physiological and microbial underpinnings of differential CH_4_ production between ruminat and non-ruminant herbivores, and the knowledge on correlations between the microbiota and CH_4_ production might be useful for targeted intervention of rumen microbiota to mitigate CH_4_ production from ruminants.

## Materials and Methods

### Animals, Diets, and Experimental Design

All experiments involving animals and the animal use protocols were approved by the Animal Care Committee of Zhejiang University (Hangzhou, China). Five 1.5-years old healthy male sheep (63.91 ± 6.18 kg BW) each with a permanent ruminal cannula were each allocated to an open circuit respiration chamber, which was constructed using aluminum frames and resin sheets, allowing animals in neighboring chambers see each other. Temperature and humidity inside the chambers were respectively maintained at 25°C and 60%. Before gas determination, both the door and the food hopper of each chamber were kept open. Fifteen 1-year old healthy male NZW rabbits (3.14 ± 0.14 kg BW) were each housed in an indoor cage (60 × 50 × 35 cm in dimensions). Both the chambers and the cages were placed in a temperature-regulated room (24–26°C) with a natural light-dark cycle (approximately 13 h of light and 11 h of dark). Both the sheep and the rabbits were fed the same diet consisting of only alfalfa hay (18.5% CP, 46.0% NDF and 33.0% ADF) and had *ad libitum* access to fresh drinking water during the whole feeding experiment of 23 days for the sheep and 24 days for the rabbits. The feeding experiment consisted of 15 days for acclimation, 7 days for sample collection, and 1 day for gas measurement.

### Measurement of Feed Intake and Digestibility

At the beginning of the experiment, the rabbits were blocked by BW (5 blocks, 3 rabbits per block), and each block was transferred to an indoor metabolism cage. The amount of feed offered and refused was recorded daily, and all feces were collected using fecal collection plates at 8:30 AM daily from individual sheep and blocks of rabbits during the 7 days of sample collection. Daily feed and orts samples were pooled by experimental unit (individual sheep and rabbit blocks). Fecal output was weighed, and 100 g wet feces were added to 10 mL 10% hydrochloric acid to preserve the samples for nitrogen analysis. All the feed and the fecal samples were dried in a forced-air oven at 65°C for 72 h, then ground through a 1 mm screen, and stored in sealed plastic containers at 4°C until analysis. Standard analysis methods (Official Methods of Analysis [AOAC], 2015) were used for analysis for dry matter (DM, method 930.15), CP (method 990.03), and ADF (method 973.18). NDF contents were analyzed following the procedure of Van Soest et al. (1991) without sodium sulfite and amylase added. The BW was weighted before measuring gas production at day 22 of the feeding experiment.

### Determination of Gas Production

Gas production was determined using open circuit chambers (1.16 m^3^ interior volume each). Each chamber was completely airtight but received a continuous air flow at 8.0 m^3^ h^-1^. Total air flow was recorded using a flow meter (model number: SY-LWD-B-20; Shi Yi Automation Equipment Co., Ltd., Hangzhou, China), and concentration of CH_4_ and CO_2_ was determined using a gas detector (model number: Photoacoustic Gas Monitor INNOVA 1412; Innova AirTech Instruments A/S, Ballerup, Denmark). The alfalfa hay diet was provided to the animals twice daily at 09:00 and 16:00 using a food hopper that was reloaded outside of the chambers via a lid without opening the whole chamber. After the sample collection period, gas production from each sheep was directly determined for 24 h at day 23 of the feeding experiment. Each block of the rabbits was transferred to a chamber at day 23 of the feeding experiment for 24 h acclimation before continuous gas measurement for 24 h at day 24. CH_4_ production was expressed as CH_4_ yield per kg of BW^0.75^, DMI, digestible NDF intake, and digestible ADF intake, while CO_2_ production was expressed as CO_2_ yield per kg of BW^0.75^.

### Collection of Ruminal and Cecal Samples

Ruminal content samples (about 50 mL each) were taken from individual sheep through their rumen cannula immediately after completing the gas determination. All the rabbits were sacrificed following euthanasia that was administered by a licensed animal technician following the procedures described by Yang et al. (2016). A quiet environment was provided to individual rabbit on a table with a slight angle to avoid stress and minimize pressure on the diaphragm. Rabbits were intravenous injected phenobarbital sodium (Sigma, Saint Louis, MO, United States) with a dose of 100 mg/kg BW. Once losing toe pinch and leg withdrawal reflex, each rabbit received ear intravenous injection of 20 mL of air. Then, cecal content samples were immediately collected (Yang et al., 2016). Briefly, each cecum was delineated into its proximal, middle, and distal segments, which were tied at the boundaries with a nylon string to prevent the cecal digesta from moving longitudinally. Each of the three cecal segments was cut separated, and its digesta content was squeezed into one 50-ml sterile Falcon tubes within 30 min of decease. One composite sample was prepared for each rabbit by combining about the same amount of digesta from each cecal segment. After immediate pH measurement using a pH meter (PB-10; Sartorius, Göettingen, Germany), the cecum samples were stored in liquid nitrogen and transported to the laboratory. Approximately 20 g of each rumen content and cecal content sample were freeze-dried for 30 h using a freeze-dryer (model number: BETA 1-8 LD; Martin Christ Gefriertrocknungsanlagen GmbH, Osterode, Germany). Each of the freeze-dried samples was crushed into fine particles manually and stored at -80°C until further analysis.

### Measurement of Volatile Fatty Acid (VFA) Concentrations

An aliquot of each ruminal and cecal sample was subjected to analysis for VFAs as described by Yang et al. (2015). Briefly, approximately 2 g of each ruminal content sample and 1 g of each cecal content sample were added to 5 mL and 3 mL sterile phosphate buffered saline (PBS, pH 7.0), respectively, and mixed, and the mixture was centrifuged at 13,000 ×*g* at 4°C for 15 min. To 1 mL of each supernatant were added 20 μL of 85% orthophosphate acid and centrifuged again as described above to obtain the final supernatant. The VFAs concentration in the supernatant was determined using a gas chromatograph (model number: GC-2010; Shimadzu Corp., Kyoto, Japan) against external standards purchased from Aladdin (China, Shanghai).

### Measurement of Microbial Enzyme Activity

The activities of CMCase, MCCase, xylanase, and pectinase of each sample was determined essentially as described previously (Wang et al., 2015), using carboxymethyl cellulose sodium (Sigma-Aldrich, Saint Louis, MO, United States), microcrystalline cellulose (Sigma-Aldrich), beechwood xylan (Sigma-Aldrich), and pectin from citrus peel (Fluka, St. Louis, MO, United States) as respective substrates, according to the dinitrosalicylic acid (DNS) method expounded by Bailey et al. (1992). Briefly, approximately 0.5 g of each freeze-dried ruminal content sample or cecal content sample was vortexed in 6 mL sterile PBS (pH 7.0), and the sample suspension was then sonicated (20 kHz, 195 W, 10 min) using a JY92-IIN Ultrasonic Cell Mixer (Ningbo Scientz, Ningbo, China) and centrifuged at 12,000 ×*g* at 4°C for 10 min. Then, 0.2 mL of the supernatant of each sample was mixed with 0.2 mL corresponding substrates (0.01 g mL^-1^ in phosphate buffer, pH 6.6) and then incubated at 39°C for 30 min. The enzyme activity was expressed as μmoL of glucose (for CMCase and MCCase), xylose (for xylanase), or D-galacturonic acid (for pectinase) released min^-1^ g^-1^ of the freeze-dried samples or their microbial crude protein (MCP).

### Measurement of Microbial Crude Protein

Approximately 0.5 g of each freeze-dried ruminal content sample or cecal content sample was vortexed in 6 mL sterile PBS buffer solution (pH 7.0) to get the microbes in the gut fluid and most of the microbes adhering to the feed particles, and the suspension was then centrifuged at 408 ×*g* for 5 min to remove protozoa and remain feed particles. Then, 1 mL of each supernatant was centrifuged at 25,000 × *g* at 4°C for 20 min. The supernatants were discarded, and the pelleted microbial cells were suspended in 3 mL of 0.25 N sodium hydroxide and heated in boiling water for 10 min to lyse the microbial cells. The cell lysate samples were centrifuged at 25,000 ×*g* for 30 min, and the supernatants were subjected to protein assay with bovine serum albumin as the standard using the Coomassie brilliant blue (CBB) method (Makkar et al., 1982). The content of MCP was expressed as mg g^-1^ freeze-dried ruminal content or cecal content samples.

### DNA Extraction and Real-Time PCR Quantification

Metagenomic DNA was extracted from 0.1 g each of freeze-dried ruminal content sample or cecal content sample using the CTAB (cetyltrimethylammonium bromide) method but with bead-beating (Gagen et al., 2010). The quality of the DNA extracts was evaluated using agarose (1%) electrophoresis, while the DNA concentration was determined using the Qubit dsDNA BR Assay Kit (Invitrogen Corporation, United States) on a Qubit 2.0 fluorometer (Invitrogen Corporation, United States). Standards for qPCR assay were prepared for individual groups of targeted microbes or targeted genes (the primers were listed in Supplementary Table S1) using cloning of PCR amplicons with a pGEM^®^T Easy kit (Promega, Shanghai, China) following the method of Koike et al. (2007). The abundance of each species or group of microbes was quantified using real-time PCR as described previously (Liu et al., 2014) and expressed as log_10_ copies of 16S rRNA gene (or 18S rRNA gene in the case of protozoa, and ITS1 in the case of fungi) per g of freeze-dried ruminal content or cecal content samples.

### Analysis of Microbiota

One amplicon library each was separately prepared for archaea and bacteria from each of the metagenomic DNA samples using the primers M86F/M448R and 515F/806R, respectively. All amplicons were pooled in equal molar ratio and sequenced using the 2 × 250 bp paired-end protocol on an Illumina MiSeq system. The raw sequences were de-multiplexed, quality-filtered, and analyzed using QIIME (v 1.9.0) (Caporaso et al., 2010). Briefly, bases from each sequencing read with a Q score less than 25 were trimmed off, then the paired reads (R1 and R2) were merged to form single sequences using the fastq-join script (Aronesty, 2011). Sequences shorter than 352 bp for archaea and 281 bp for bacteria were discarded, and the primers were further trimmed off. Chimera checking was performed using the ChimeraSlayer algorithm (Haas et al., 2011). The quality-checked sequences were clustered into species-equivalent operational taxonomic units (OTUs) by comparison to the Greengenes database 13.5 (DeSantis et al., 2006) using the open-reference OTU picking option (pick_open_reference_otus.py). The OTUs were taxonomically classified by comparison to the Greengenes database 13.5. Minor OTUs were filtered out if they were each represented by less than 0.005% of the total sequences (Bokulich et al., 2013) or appeared in less than 60% of each experimental animal species. The sequences of each sample were rarefied to the same number (46,609 sequences/sample for archaea and 19,170 sequences/sample for bacteria) before diversity analysis. Alpha diversity measurements including Chao1 richness estimate, Shannon diversity index, and observed number of OTUs were calculated for each sample. The microbiota were compare as beta diversity using the distance matrices generated from weighted UniFrac analysis (Lozupone and Knight, 2005) and principal coordinates analysis (PCoA). The raw sequence data were deposited in the Sequence Read Archive of NCBI under accession no. SRP108266.

### Statistical Analysis

Statistical analysis of the data was performed using one-way ANOVA, with means separation using *t*-test at the level of significance of 0.05 using the SAS software package (SAS Institute Inc., 2000). Pearson correlation coefficients were calculated to examine the correlation between animal performances and relative abundance of microbial groups. The data were expressed as Mean ± SD in the Tables.

## Results

### Feed Digestibility and Gas Yields

**FIGURE 1:**
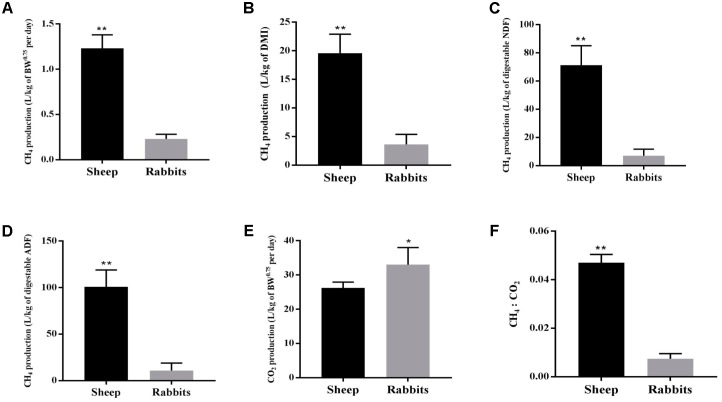
Yields of CH_4_ and CO_2_ and their ratio of the sheep (*n* = 5) and the rabbits (*n* = 15). CH_4_ yields per unit of metabolic body weight **(A)**, dry matter intake **(B)**, digestible NDF **(C)**, and digestible ADF **(D)**; CO_2_ yields per unit of metabolic body weight **(E)** and CH_4_ : CO_2_ ratio **(F)** of the sheep and the rabbits. ^∗∗^*P* < 0.01; ^∗^*P* < 0.05.

The two species of animals used in this study consumed a similar amount of feed (DM) per unit of BW^0.75^ daily (**Table 1**). However, the sheep had a higher apparent digestibility of DM, NDF, and ADF than the rabbits (*P* < 0.05). Each of the sheep emitted substantially more CH_4_ than each rabbit per day per unit of BW^0.75^, DMI, digestible NDF, or digestible ADF (**Figures 1A–D**). Per unit of BW^0.75^, however, the sheep emitted less CO_2_, resulting in a 6.4 times higher CH_4_ to CO_2_ ratio than the rabbits (**Figures 1E,F**).

**Table 1:** Body weight, feed dry matter intake, and digestibility of the sheep (*n* = 5) and the rabbits (*n* = 15).

Parameter	Units	Sheep	NZW rabbits	*P*-value
Body weight	kg	63.91 ± 6.18	3.14 ± 0.13	<0.01
	kg^0.75^	22.59 ± 1.65	2.36 ± 0.075	<0.01
Dry matter intake	g/d/kg of BW^0.75^	63.42 ± 3.34	67.66 ± 15.12	0.56
Apparent digestibility	%			
Dry matter		68.91 ± 1.76	57.76 ± 7.97	0.02
NDF		60.05 ± 3.09	41.35 ± 11.83	<0.01
ADF		59.28 ± 3.15	38.26 ± 13.55	<0.01

### Fermentation Characteristics

The main fermentation characteristics, including pH, concentrations of VFA, and their molar proportions, in the sheep rumen and the rabbit cecum are presented in **Table 2**. The pH in the sheep rumen was more than 1 unit higher (*P* < 0.01) than that in the rabbit cecum. No significant difference (*P* = 0.16) in total VFA concentration was observed between the two digestive organs. However, a much lower propionate concentration was seen in the rabbit cecum than in the sheep rumen. The two digestive organs also differed (*P* ≤ 0.01) in molar proportions of VFA, with the rabbit cecum having a higher value for acetate and butyrate but a lower value for propionate. The acetate to propionate (A: P) ratio in the rabbit cecum was more than threefold greater than that in the sheep rumen.

**Table 2:** Fermentation characteristics of the sheep rumen (*n* = 5) and the rabbit cecum (*n* = 15).

Measurements	Units	Sheep rumen	Rabbit cecum	*P*-value
pH		7.11 ± 0.17	5.82 ± 0.25	<0.01
Concentration	mmol/kg			
Total VFA		67.15 ± 10.54	55.70 ± 12.97	0.16
Acetate		51.73 ± 7.90	46.37 ± 10.78	0.40
Propionate		9.12 ± 1.50	2.39 ± 0.45	<0.01
Butyrate		6.31 ± 1.65	6.95 ± 1.94	0.59
Molar proportion	%			
Acetate		77.07 ± 1.79	83.26 ± 1.54	<0.01
Propionate		13.58 ± 0.97	4.33 ± 0.26	<0.01
Butyrate		9.34 ± 1.35	12.41 ± 1.61	0.01
Acetate/Propionate		5.70 ± 0.50	19.34 ± 1.07	<0.01

### Microbial Crude Protein Yields and Enzymes Activity

The rabbit cecal content had a higher (*P* < 0.01) concentration of MCP than the sheep rumen content (**Table 3**). A higher (*P* < 0.01) activity of CMCase, MCCase, and pectinase was observed in the rabbit cecum than in the sheep rumen either per g content or mg MCP. Xylanase activity was similar between the two digestive organs (**Table 3**).

**Table 3:** Microbial crude protein (MCP) and enzyme activities in the sheep rumen (*n* = 5) and the rabbit cecum (*n* = 15).

Measurements	Units	Sheep rumen	Rabbit cecum	*P*-value
MCP	mg/g content	24.01 ± 2.78	30.76 ± 2.03	<0.01
Absolute enzyme activities	U/g content			
CMCase^1^		2.00 ± 0.30	4.20 ± 0.48	<0.01
MCCase^2^		2.06 ± 0.16	5.86 ± 0.43	<0.01
Xylanase		0.050 ± 0.02	0.052 ± 0.01	0.88
Pectinase		8.80 ± 1.15	20.40 ± 3.29	<0.01
Specific enzyme activities	U/mg MCP			
CMCase^1^		0.086 ± 0.018	0.14 ± 0.015	<0.01
MCCase^2^		0.088 ± 0.011	0.19 ± 0.015	<0.01
Xylanase		0.0021 ± 0.001	0.0017 ± 0.001	0.33
Pectinase		0.37 ± 0.038	0.66 ± 0.069	<0.01

*^1^Carboxymethyl cellulase. ^*2*^Microcrystalline cellulose cellulase.*

### Abundance of Select Microbes and Genes Involved in Hydrogen Metabolism

**FIGURE 2:**
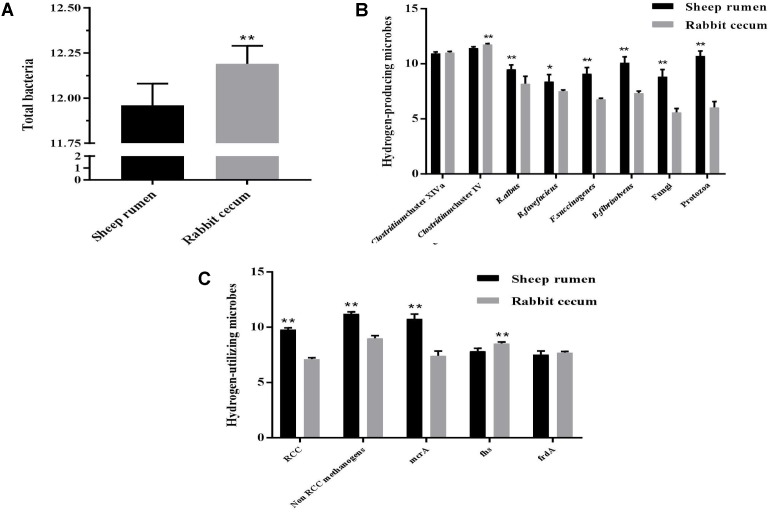
Abundance of select microbes in the sheep rumen (*n* = 5) and the rabbit cecum (*n* = 15). **(A)** Total bacteria; **(B)** Select hydrogen-producing microbes; **(C)** Select hydrogen-utilizing microbes. Abundance was expressed as log10 copies of target gene/g freeze-dried digesta sample. Target gene: 16S rRNA gene for bacteria and methanogens (except for *mcr*A); ITS1 for fungi; 18S rRNA for protozoa; *mcr*A, methyl-CoA reductase α subunit gene; *fhs*, Formyltetrahydrofolate synthetase gene; *frd*A, Fumarate reductase gene α subunit gene. ^∗∗^*P* < 0.01; ^∗^*P* < 0.05.

The total bacterial population (log_10_ 16S rRNA gene copies/g sample) was larger in the rabbit cecum than in the sheep rumen (**Figure 2A**). The abundance of *Ruminococcus albus*, *R. flavefaciens*, *Fibrobacter succinogenes*, and *Butyrivibrio fibrisolvens* was greater in the sheep rumen than in the rabbit cecum. The same holds true for the abundance of fungi and protozoa. The abundance of *Clostridium* Cluster XIVa was similar between the two digestive organs, while that of *Clostridium* Cluster IV was greater in the rabbit cecum than in the sheep rumen (**Figure 2B**). The copy number of *mcr*A gene per g sample was greater in the sheep rumen than in the rabbit cecum, while that of *fhs* gene and *frd*A gene was smaller (**Figure 2C**). The sheep rumen had a greater abundance of RCC methanogens and non-RCC methanogens (including *Methanobrevibacter*, *Methanomicrobium*, *Methanobacterium*, *Methanomicrococcus*, and *Methanosphaera*) than the rabbit cecum. The archaea : bacteria ratio differed between the two different digestive organs, 0.089 in the sheep rumen and 2.30E-05 in the rabbit cecum.

### Diversity, Species Richness, and Composition of Archaeal Microbiota

**FIGURE 3:**
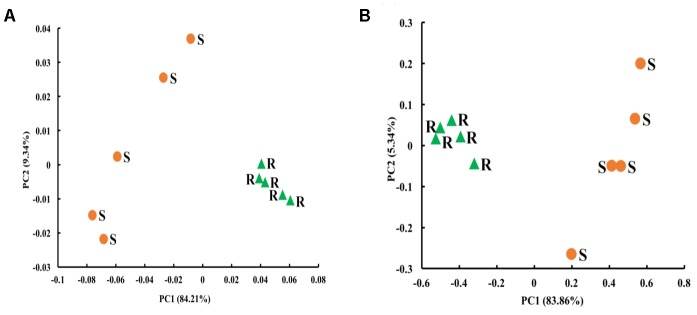
Principal coordinates analysis (PCoA) plots in the sheep rumen (*n* = 5) and the rabbit cecum (*n* = 5 blocks). **(A)** Archaeal microbiota; **(B)** Bacterial microbiota. The PCoA were based on distance matrices generated using weighted UniFrac. S, sheep rumen samples; R, rabbits cecal samples.

The Chao1 richness estimate was similar between the two digestive organs, but the rabbit cecum had a lower Shannon diversity index and Simpson index of diversity than the sheep rumen (**Table 4A**). The archaeal microbiota of the two digestive organs clustered separately along the PC1 that explained greater than 84% of total variation. The rabbit cecal archaeal microbiotas clustered relatively tightly, while those of the sheep rumen quite scattered along PC2 that explained less than 10% total variation (**Figure 3A**).

**Table 4:** Alpha diversity measurements of archaeal **(A)** and bacterial **(B)** microbiota of the sheep rumen (*n* = 5) and the rabbit cecum (*n* = 5 blocks).

Measurements	Sheep rumen	Rabbits cecum	*P*-value
**(A)**
Number of sequences per sample	50546 ± 2681	110748 ± 14572	
Observed species	134.70 ± 8.90	138.72 ± 23.99	0.73
Chao1	142.47 ± 7.88	156.00 ± 14.44	0.10
Shannon index	3.20 ± 0.33	2.31 ± 0.26	<0.01
Simpson	0.80 ± 0.08	0.65 ± 0.04	0.01
**(B)**
Number of sequences per sample	34284 ± 7253	32452 ± 2983	
Observed species	721.80 ± 36.31	647.38 ± 12.15	<0.01
Chao1	768.51 ± 32.43	687.28 ± 20.27	<0.01
Shannon index	7.38 ± 0.25	6.60 ± 0.10	<0.01
Simpson index	0.98 ± 0.01	0.96 ± 0.01	<0.01

**FIGURE 4:**
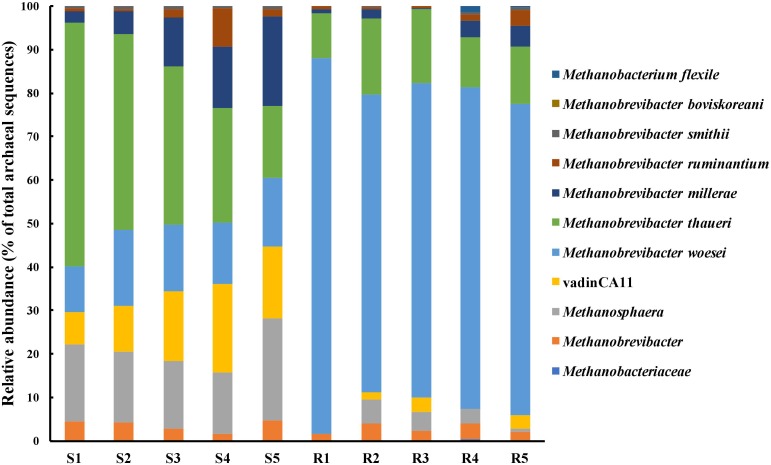
Comparison of the relative abundance of species and genera of methanogens identified in the two digestive organs. S1–S5, the five sheep; R1–R5, the five blocks of rabbits.

Approximately 99.7% of the sequences obtained from both the digestive organs were assigned to known archaeal genera (**Figure 4**). More than 95% of the archaeal sequences from the rabbit cecum were assigned to the genus *Methanobrevibacter*, while the archaeal sequences from the sheep rumen were assigned to *Methanobrevibacter* (68.3%), *Methanosphaera* (17.3%), and unidentified achaeon vadinCA11 (14.2%). The two digestive organs shared 120 archaeal OTUs besides their unique archaeal OTUs. The 30 OTUs unique to the sheep rumen were assigned to *Methanobrevibacter* (8 OTUs), vadinCA11 (10 OTUs), and *Methanosphaera* (8 OTUs), together accounting for 3.6% of total archaeal sequences identified therein. Of the 59 OTUs only found in the rabbit cecum, 53 were classified to *Methanobrevibacter*, and these 53 *Methanobrevibacter* OTUs accounted for only 1.1% of total archaeal sequences identified in the rabbit cecum. A significant portion of the archaeal sequences was assigned to known species, with 36.3% assigned to ^∗∗∗^*M. thaueri*, 14.6% to *M. woesei*, and 10.8% to *M. millerae* for the sheep rumen sequences, while for the rabbit cecal sequences, 74.6% to *M. woesei*, and 13.9% to *M. thaueri*.

### Diversity, Species Richness, and Composition of Bacterial Microbiota

The number of OTUs, Chao1 richness estimate, Shannon diversity index, and Simpson index of diversity in the sheep rumen were all significantly greater than those in the rabbit cecum (**Table 4B**). When compared using weighted UniFrac distance, the rabbit cecal bacterial microbiotas were separated, as a tight cluster, from those of the sheep rumen along the PC1 that explained greater than 83% of total variation (**Figure 3B**). The sheep rumen bacterial microbiotas of the five sheep exhibited considerable scattering along PC2, but it only explained less than 6% of total variation.

**FIGURE 5:**
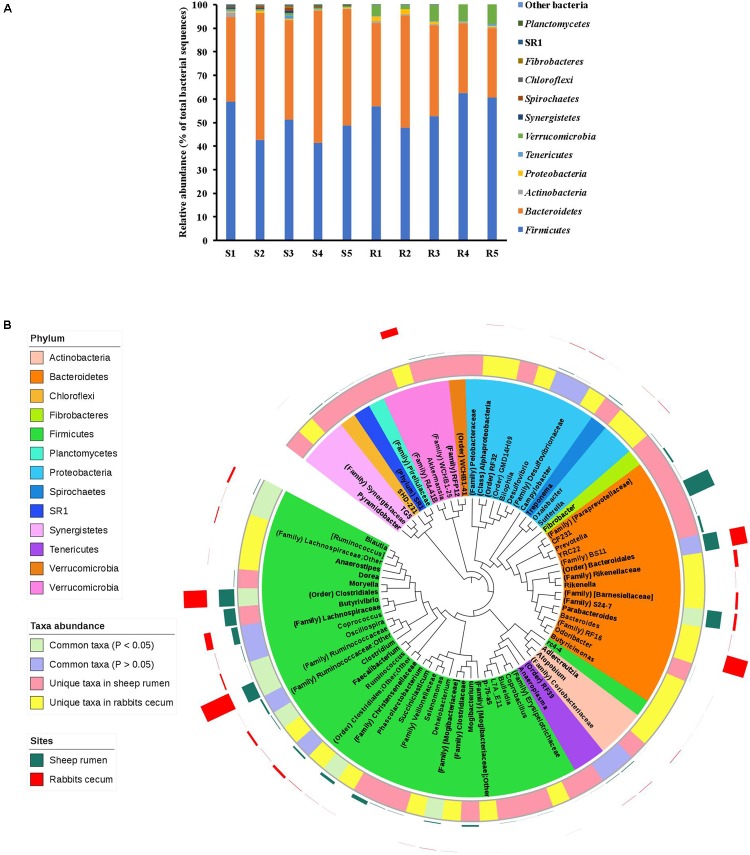
Comparison of the bacterial microbiota in the sheep rumen and the rabbit cecum. **(A)** The relative abundance of bacterial phyla; **(B)** A neighbor-joining phylogenetic tree of the representative sequences of the OTUs that were graphically displayed in the Interactive Tree of Life. S1–S5, the five sheep; R1–R5, the five blocks of rabbits.

Almost all the sequences obtained from both the sheep rumen and the rabbit cecum were assigned to known bacterial phyla, with *Firmicutes* (48.7 vs. 56.1%) and *Bacteroidetes* (47.4 vs. 36.1%) being represented by more sequences than other phyla (**Figure 5A**). Seven bacterial phyla were identified in both the sheep rumen and the rabbit cecum, which included, in addition to the above two predominant phyla, *Actinobacteria*, *Proteobacteria*, *Tenericutes*, *Verrucomicrobia*, and *Synergistetes*. However, another five bacterial phyla, i.e., *Spirochaetes*, *Chloroflexi*, *Fibrobacteres*, *Planctomycetes*, and candidate phylum SR1 were only found in the sheep rumen. Of the common bacterial phyla, the relative abundance of *Verrucomicrobia* was significantly greater in the rabbit cecum than in the sheep rumen (5.7 vs. 0.6%, *P* < 0.01).

In total, 840 and 728 OTUs were respectively observed in the sheep rumen and the rabbit cecum, and only 51 OTUs were found in both digestive organs. These common OTUs were assigned, at the lowest taxonomic rank, to the candidate order RF39 (3 OTUs), *Clostridiales* (23 OTUs), *Bacteroidales* (2 OTUs), candidate family S24*-7* (1 OTUs), *Ruminococcaceae* (7 OTUs), *Lachnospiraceae* (4 OTUs), *Ruminococcus* (3 OTUs), *Oscillospira* (3 OTUs), *Blautia* (3 OTUs), and *Bacteroides* (1 OTUs). These common OTUs represented 9.6% and 16.9% of the total bacterial sequences in the sheep rumen and the rabbit cecum, respectively.

The OTUs found in the sheep rumen and the rabbit cecum was assigned to 49 and 45 lowest possible taxa, respectively. However, only 25 (for the sheep rumen) and 24 (for the rabbit cecum) of them are recognized genera. The two digestive organs shared 18 common taxa, leaving 31 taxa being unique in the sheep rumen and 27 taxa found only in the rabbit cecum (**Figure 5B**). The predominant taxa (each represented by >1.0% of total bacterial sequences in at least 3 of the 5 experiment units) common to the sheep rumen and the rabbit cecum included *Bacteroidales* (11.1 vs. 12%, *P* = 0.83), *Lachnospiraceae* (4.9 vs. 5.3%, *P* = 0.82), and *Ruminococcus* (1.4 vs. 1.9%, *P* = 0.24). A small number of taxa had different relative abundance between the sheep rumen and the rabbit cecum, with *Ruminococcaceae* (10.1 vs. 24.1%, *P* < 0.01), *Clostridiales* (12 vs. 18%, *P* = 0.03), *Oscillospira* (0.1 vs.1.2%, *P* < 0.01), *Blautia* (0.4 vs. 1.7%, *P* = 0.01), and *Clostridium* (0.5 vs. 1.7%, *P* = 0.01) being less predominant, while *Christensenellaceae* (2.2 vs. 0.1%, *P* < 0.01) and candidate family S24-7 (11.3 vs. 2.1%, *P* = 0.02) being more predominant in the sheep rumen than in the rabbit cecum. The major unique taxa (with a relative abundance of >1.0% in at least 3 of the 5 experiment units) in the sheep rumen included *Prevotella* (21.3%), *Butyrivibrio* (9%), *Succiniclasticum* (3%), candidate family BS11 (1.7%), candidate family [*Paraprevotellaceae*] (1.4%), and *Mogibacterium* (1.3%). *Bacteroides* (16.9%), *Akkermansia* (5.7%), *Rikenellaceae* (2.9%), and candidate family [*Barnesiellaceae*] (2%) were the major taxa unique to the rabbit cecum.

### Pearson Correlation Between Chemical Parameters and Dominant Bacterial Taxa

**FIGURE 6:**
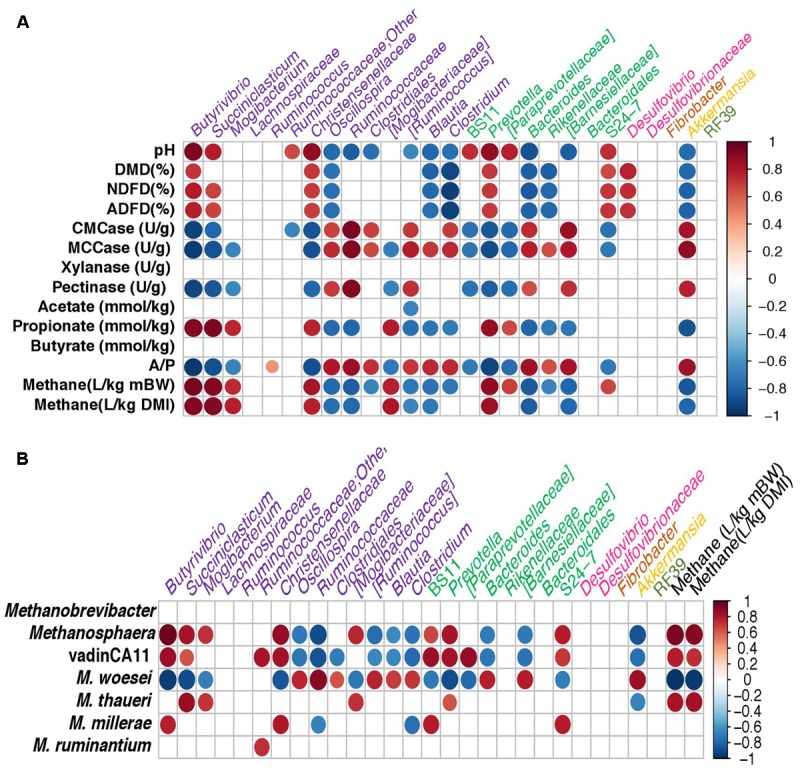
Pearson correlation between **(A)** chemical parameters and the relative abundance of bacterial taxa; **(B)** Archaeal taxa and bacterial taxa in relative abundance for the sheep rumen (*n* = 5) and the rabbit cecum (*n* = 5 blocks) in combination. Taxa were included in the matrix only if they were found in at least 3 of the five sheep or five rabbit blocks and they were each represented by at least 1.0% of the total bacterial sequences. Size of the filled circle reflects the strength of the correlations. Only significant correlation (*P* < 0.05) were shown with colors. The scale colors denote the direction of correlation (1 to –1). The taxa assigned to different phyla were color coded: purple, *Firmicutes;* green, *Bacteroidetes;* pink, *Proteobacteria;* brown, *Fibrobacteres;* yellow, *Verrucomicrobia;* and olive*, Tenericutes*.

Pearson correlation coefficients were calculated to reveal correlations between the animal phenotypic data and the predominant bacterial taxa (**Figure 6A**). The relative abundance of *Butyrivibrio*, *Prevotella*, *Succiniclasticum*, *Mogibacterium*, *Christensenellaceae*, candidate family [*Mogibacteriaceae*], and candidate family S24-7 appeared to be positively correlated (*P* < 0.05) with both CH_4_ yield and feed digestibility, whereas that of *Oscillospira*, *Ruminococcaceae*, *Clostridiales*, candidate genus [*Ruminococcus*], *Blautia*, *Clostridium*, *Bacteroides*, *Rikenellaceae* and *Akkermansia* was negatively (*P* < 0.05) correlated with these two measurements. A positive correlation was also seen between A:P ratio and the relative abundance of some taxa, including *Oscillospira*, *Ruminococcaceae*, *Clostridiales*, candidate genus [*Ruminococcus*], *Blautia*, *Clostridium*, *Bacteroides*, *Akkermansia*, and *Rikenellaceae*.

### Pearson Correlation Between the Archaeal Taxa and Bacterial Taxa in Relative Abundance

Pearson correlation coefficients were calculated to reveal correlations between the relative abundance of archaea and bacteria (**Figure 6B**). The relative abundance of *M. thaueri* appeared to be positively correlated (*P* < 0.05) with *Succiniclasticum*, *Mogibacterium*, candidate family [*Mogibacteriaceae*], *Prevotella*, and CH_4_ yields, and was negatively (*P* < 0.05) correlated with *Akkermansia*. The relative abundance of *Methanosphaera* and unidentified achaeon vadinCA11 appeared to be positively correlated (*P* < 0.05) with *Butyrivibrio*, *Succiniclasticum*, *Christensenellaceae*, candidate family [*Mogibacteriaceae*], BS11, *Prevotella*, S24-7, as well as CH_4_ yields, whereas were negatively (*P* < 0.05) correlated with the relative abundance of *Oscillospira*, *Ruminococcaceae*, candidate genus [*Ruminococcus*], *Blautia*, *Clostridium*, *Bacteroides*, and *Akkermansia*. The opposite correlations of *M. woesei* with both CH_4_ yields and those bacteria were showed.

## Discussion

Both ruminants and non-ruminant herbivores emit CH_4_, but the former emits much more CH_4_ than the latter (Franz et al., 2010, 2011; Cabezas Garcia, 2017). It has been speculated that such difference in CH_4_ emission is probably attributable primarily to the differences in the microbiota of the rumen and the hindgut of non-ruminant herbivores (Yang et al., 2016). However, the microbiological peculiarity for the different CH_4_ emissions by these two groups of herbivores is largely unknown. Identification of these responsible microbes and the relationship to CH_4_ emission and the fermentation characteristics of the rumen and the cecum will help understand the factors that affect CH_4_ production in the rumen and develop dietary strategies to effectively mitigate CH_4_ emission from ruminants. In the present study, we comparatively characterized the microbiota and the fermentation characteristics in the rumen of sheep and the cecum of rabbits when fed the same diet. This approach allowed us to quantitatively determine and compare CH_4_ production by a representative species of ruminants and non-ruminants on the basis of feed intake, feed digestibility, and metabolic BW^0.75^. This approach overcomes the limitation of using the same ruminant animal in which CH_4_ emission from the rumen and large intestines cannot be independently determined, and the two digestive organs received different fermentation substrates.

The rabbits produced no more than ¼ of the amount of CH_4_ produced by the sheep when compared on per unit of BW^0.75^, DMI, digestible NDF or ADF. Franz et al. (2010, 2011) proposed a linear relationship between BW and CH_4_ production by both ruminants and non-ruminants. However, the magnitude of different CH_4_ outputs between the sheep and the rabbits probably suggests physiological and microbiological peculiarities of these two digestive organs. First, the pH inside the rabbit cecum was nearly 1.3 units lower than that in the sheep rumen. It is well documented that methanogenesis is inhibited at low pH, as exemplified by no CH_4_ production at pH 5.5 or below in *in*-*vitro* cultures inoculated with rumen fluid from roughage-fed cows (Russell, 1998). Thus, the lower pH in the rabbit cecum (pH 5.8) than in the sheep rumen (pH 7.1) is probably a major chemical factor attributable to the low CH_4_ output from the rabbits. The rumen receives a large volume of saliva (about 1.31 L of saliva is secreted from one parotid gland per day for an adult sheep), which buffers the acidity from VFA (McDougall, 1948), while cecum receives no saliva. The lack of saliva secretion to the cecum is probably one of the reasons for the lower pH in the rabbit cecum than in the sheep rumen. Second, the Eh in the rabbit cecum ranges from -160 to -210 mV (Kimsé et al., 2010; Michelland et al., 2010), while the rumen Eh ranges from -268 to -318 mV (Mathieu et al., 1996). We did not analyze the Eh in the present study, but it should be within the above range. The tubiform and small in diameter of the rabbit cecum may explain its relatively higher Eh. Apparently, the Eh of the rabbit cecum is not optimal for hydrogenotrophic methanogenesis, which requires -238 mV (Cord-Ruwisch et al., 1988). Indeed, CH_4_ production in a *Methanothermobacter thermautotrophicus* culture was suppressed at Eh higher than -200 mV (Hirano et al., 2013). Future research using *in vitro* cultures of both digestive organs is warranted to verify if Eh is a primary factor determining the different CH_4_ production in the rumen and the cecum. The Eh of *in vitro* cultures can be regulated using bioelectrochemical systems that can control Eh without using oxidative and reducing agents (Hirano et al., 2013). Furthermore, digesta passage rate through the rumen has been found reversely correlated with CH_4_ production therein (Janssen, 2010; Goopy et al., 2014; Shi et al., 2014). The cecum is a tubiform tract, while the rumen is a large sac. Such structural difference can causes a faster digesta passage rate through the rabbit cecum than through the sheep rumen, contributing to the less CH_4_ output from the rabbits than from the sheep. This premise is consistent with the high passage rate and low CH_4_ production by kangaroos, a group of tubiform foregut fermenters (Von Engelhardt et al., 1978).

In the present study, we analyzed the diversity and structure of the archaeal microbiota and quantified the abundance of methanogens to understand the archaeal underpinning of the different CH_4_ yields between the two digestive organs. The rabbit cecum had a lower abundance of RCC methanogens, non-RCC methanogens, and total methanogens (as quantified as *mcr*A gene copies/g sample) than the sheep rumen. This is consistent with the finding in sheep (Popova et al., 2013), reindeer (Wedlock et al., 2013), and Chinese roe deer (Li et al., 2014), in which a greater abundance of methanogens was found in the rumen than in the cecum. The low abundance of methanogens in the rabbit cecum may be explained partially by the low pH, probably a higher *Eh*, and a greater passage. These three factors might have directly decreased methanogenesis in the rabbit cecum. Although the abundance of methanogens in the rumen does not necessarily linearly correlate to CH_4_ output (Danielsson et al., 2012; Patra and Yu, 2014), the greater abundance of methanogens in the sheep rumen than in the rabbit cecum corroborates the more CH_4_ produced by the former than by the latter.

The two digestive organs each harbored a distinct archaeal microbiota, with *M. woesei* being the dominant species in the rabbit cecum, whereas the sheep rumen containing *M. thaueri* as the most predominant known species followed by *M. millerae* and *M. woesei.* Based on a literature search of the Pubmed, only one study has analyzed the archaeal microbiota in the cecum of rabbits, and *M. woesei* was represented by more cloned 16S rRNA gene sequences than other species (Kušar and Avguštin, 2010). *M. ruminantium*, one of the two species (*M. ruminantium* and *M. Olleyae*) in the *Methanobrevibacter* RO clade (Janssen and Kirs, 2008; Kittelmann et al., 2013), was found in both the digestive organs at low relative abundance and low correlation with CH_4_ yield. The RO clade was found associated with low CH_4_ yield in the rumen (Danielsson et al., 2012). The dominance of *M. woesei*, which has only been reported in the chicken cecum (Saengkerdsub et al., 2007) other than rabbit cecum, is of further research interest. It is also interesting to note that *M. thaueri* and *M. millerae*, two of the four species (*M*. *smithii*, *M*. *gottschalkii*, *M*. *Millerae*, and *M*. *thaueri*) in the *Methanobrevibacter* SGMT clade (Janssen and Kirs, 2008; Kittelmann et al., 2013), were more predominant in the sheep rumen than in the rabbit cecum. The *Methanobrevibacter* SGMT clade, which possesses methyl coenzyme M reductase isozymes Mcr I and Mcr II and are competitive at high hydrogen concentrations (Leahy et al., 2010), has been reported to have a positive association with CH_4_ emissions from ruminants (Tapio et al., 2017). The positive correlation between *M. thaueri* and CH_4_ yield was found in the present study. It is not known if the differential predominance of these methanogen species is one explanation of the different CH_4_ production seen between the two animal species. Several studies revealed a strong correlation between CH_4_ yields and archaea: bacteria ratio (Wallace et al., 2014), and a similar finding was found in the present study. However, the previous study indicated that it was gene expression rather than gene abundance of methanogens that was strongly correlated with CH_4_ yields from sheep (Shi et al., 2014). Metatranscriptomic studies will help determine the contribution of each methanogen species to the overall CH_4_ yield in these two digestive organs.

Methane is produced by methanogens, but other members of the microbiota can determine or profoundly affects the rate and yield of methanogenesis (Kittelmann et al., 2014; Danielsson, 2016). In the present study, we characterized the bacterial microbiota for diversity and structure and quantified the population of microbes that produce hydrogen, including anaerobic fungi, protozoa, select hydrogen-producing bacteria, and acetogens to help understand the role and significance of these microbes in determining the different CH_4_ yields between the two digestive organs. Expectedly, the sheep rumen and the rabbit cecum differed in the communities of the above microbes. Different microbiota were also reported between the rumen and the cecum of growing bulls (Popova et al., 2017), reindeer (Wedlock et al., 2013), and Chinese roe deer (Li et al., 2014). Such difference may be attributable to the combined effect of a host of factors, including pH, saliva (present in the rumen but not in cecum), passage rate, nutrients (lack of non-structural carbohydrates and protein due to digestion and absorption in the foregut), mixing, Eh (higher in the cecum), mucosa and antimicrobial peptides (both present in the rabbit cecum but not in the sheep rumen).

Hydrogen-producing microbes provide the reducing power for hydrogenotrophic methanogenesis, and indeed, sheep producing more hydrogen also produced more CH_4_ than those that produced less hydrogen (Kittelmann et al., 2014). As determined by qPCR, the sheep rumen did have a greater abundance of *R. albus*, *R. flavefaciens*, *B. fibrisolvens*, fungi, and protozoa, all of which can produce hydrogen during feed fermentation, than the rabbit cecum. Combined analysis of the bacterial microbiota also revealed correlations between several animal phenotypic measurements and individual bacterial groups. Among the bacterial genera whose relative abundance was strongly and positively correlated with CH_4_ yield, *Prevotella* and related bacteria, *Butyrivibrio*, and *Succiniclasticum* are unique and/or predominant hydrogen-producing bacteria in the rumen (Leahy et al., 2013). The bacterial genera whose relative abundance appeared to be negatively correlated with CH_4_ yield are either acetogens (e.g., *Blautia*) (Müller and Frerichs, 2013), butyrate producers (e.g., *Oscillospira*) (Gophna et al., 2017), or succinate producers (e.g., *Bacteroides*) (Song et al., 2015). In the rumens of high methane-emitting sheep, members of *Ruminococcaceae* and *Lachnospiraceae* were found at higher relative abundance, while the rumens of low methane-emitting sheep were enriched with *Erysipelotrichaceae*, especially *Sharpea* spp. (Kamke et al., 2016). However, the low correlation between CH_4_ yield and the relative abundance of *Ruminococcus* and *Lachnospiraceae* were found when comparing the two different the digestive organs in the present study. The relationship between the abundance of *F. succinogenes* and CH_4_ production cannot be explained also, nor the contribution of *Clostridium* cluster IV. It should be noted that the correlation of a few bacterial genera with CH_4_ yield might be due to their occurrence in only one of the two digestive organs, such as *Mogibacterium* and *Succiniclasticum* that were only detected in the rumen, and *Akkermansia* that was only detected in the large intestines. Nevertheless, the positive correlation between CH_4_ yield and the relative abundance of *Mogibacterium*, which does not ferment carbohydrate (Nakazawa et al., 2015), is consistent with its ability to produce phenylacetate, a metabolite that is needed for the degradation of cellulose by some *R. albus* strains (Morrison et al., 1990).

Constant hydrogen disposal is essential for sustained fermentation in the rumen and the large intestines (Moss et al., 2010). Thus, alternative hydrogen utilization pathways must exist in the rabbit cecum. We analyzed two genes, *fhs* and *frd*, involved in two different [H]-utilizing pathways to understand the alternative hydrogen utilization potential in the two digestive organs. The rabbit cecum had a higher abundance of *fhs*, which encodes formyltetrahydrofolate synthetase, a key enzyme in the homoacetogenesis, consistent with the lower CH_4_ production and higher molar proportion of acetate therein. A strong positive correlation was also found between A: P ratio and some bacterial taxa, including some taxa of *Clostridiales*, *Lachnospiraceae*, *Ruminococcaceae*, and *Blautia*, all of which contain known acetogens (Yang et al., 2016). All these results suggest acetate is not just a hydrogen donor, but a hydrogen sink. Indeed, homoacetogenesis might be predominant in the rabbit cecum than in the sheep rumen. The dominance of homoacetogenesis has been reported in some tubiform gut ecosystems, such as rabbits cecum (Yang et al., 2016), kangaroos foregut (Gagen et al., 2010), and termite hindgut (Ottesen and Leadbetter, 2011). It has been suggested that pH might determine the predominance of hydrogen disposal pathways, with relatively neutral pH favoring methanogens and acidic pH favoring acetogens (Gibson et al., 1990). The relatively lower pH in the cecum of rabbits probably suppress hydrogenotrophic methanogenesis, allowing homocaetogenesis to increase. It is interesting to note that more CO_2_ was produced from the rabbits than from the sheep per kg of BW^0.75^. It is likely that less CO_2_ is consumed during homoacetogenesis in the cecum than during methanogenesis in the sheep rumen. This premise is consistent with the less thermodynamic feature of homoacetogenesis than hydrogenotrophic methanogenesis and the higher *Ks* of the former (Kohn and Boston, 2000). The lower pH in the rabbit cecum could also decrease the CO_2_ solubility and thus more CO_2_ emission.

Surprisingly, significantly greater activities of CMCase, MCCasse, and pectinase were detected in the rabbit cecum than in the sheep rumen, suggesting an enrichment of fibrolytic and pectinolytic microbes in the rabbit cecum. Along with the significantly different bacterial microbiota structures between the sheep rumen and the rabbit cecum, our findings indicate that rabbit cecum probably harbors novel and uncharacterized cellulolytic bacteria and glycoside hydrolases. These novel microbes and enzymes can be identified in future studies using functional metagenomics and transcriptomics.

## Conclusion

The present study demonstrates that different methane production between the sheep and the rabbits can be explained by the different physiological environments of their respective digestive organs and the microbiota residing therein. Lower abundance of hydrogen-producing microbes (bacteria, fungi, and protozoa) and methanogens, and increased homoacetogenesis as an alternative hydrogen utilization pathway in the rabbit cecum might result in lower CH_4_ yield from the rabbits. The cecum of rabbits is potentially a rich resource to fibrolytic bacteria and hence novel cellulolytic enzymes. Future studies using functional approaches, such as functional metagenomics and transcriptomics, will help reveal the potential and functionality of metabolic pathways involved in fiber digestion, methanogenesis, and acetogenesis and help develop new strategies to achieve effective CH_4_ mitigation for ruminal livestock.

## Author Contributions

JW, LM, and JL conceived and designed the study. LM performed both animal feeding and laboratory experiments, analyzed the sequencing data, interpreted the data, prepared the figures and tables, and wrote the manuscript. ZY and BY helped analyzing the sequencing data. XH and YL participated in the animal feeding experiments. ZY and JW helped interpret the data and write and revise the paper. All authors read and approved the final manuscript.

## Conflict of Interest Statement

The authors declare that the research was conducted in the absence of any commercial or financial relationships that could be construed as a potential conflict of interest.

## Abbreviations

A: acetate
ADF: acid detergent fiber
BW: body weight
BW^0.75^: metabolic body weight
CMCase: carboxymethyl cellulase
CP crude protein DM: dry matter
DMI: dry matter intake
Eh: redox potential
*fhs*: formyltetrahydrofolate synthetase gene
*frd*A: fumarate reductase gene α subunit
MCCase: microcrystalline cellulose cellulase
MCP: crude microbial protein
*mcr*A: methyl CoM reductase gene α subunit
NDF: neutral detergent fiber
NZW rabbit: New Zealand white rabbit
P: propionate
RCC: rumen cluster C
VFA: volatile fatty acid
