# Supplementary material for: Comparative Analysis of the Microbiota Between Sheep Rumen and Rabbit Cecum Provides New Insight Into Their Differential Methane Production
Source: Front Microbiol. 2018 Mar 27;9:575. doi: 10.3389/fmicb.2018.00575 (PMC5890152; doi:10.3389/fmicb.2018.00575)
Supplement: Supplementary file 1 [file Table_1.DOCX]

***Supplementary Material***

**Comparative Analysis of the Microbiome between Sheep Rumen and Rabbit Cecum Provides New Insight into their Differential Methane Production**

**Lan Mi^*^, Bin Yang, Xialu Hu, Yang Luo, Jianxin Liu, Zhongtang Yu, Jiakun Wang**

**^*^Correspondence:** Dr. Jiakun Wang: [jiakunwang@zju.edu.cn](mailto:jiakunwang@zju.edu.cn) Dr. Zhongtang Yu: yu.226@osu.edu

**Supplementary Table 1.** Primer sets used in this study

| Target species/genes | Primer sequences | | Product size (bp) | PCR efficiency (%) | References |
| --- | --- | --- | --- | --- | --- |
| Total bacteria | F | CGGCAACGAGCGCAACCC | 143 | 104 | Denman and McSweeney, 2006 |
|  | R | CCATTGTAGCACGTGTGTAGCC |  |  |  |
| *Clostridium* cluster XIVa | F | CGGTACCTGACTAAGAAGC | 429 | 100 | Ramirez-Farias et al., 2008 |
|  | R | AGTTTYATTCTTGCGAACG |  |  |  |
| *Clostridium* cluster IV | F | TTAACACAATAAGTWATCCACCTGG | 314 | 105 | Ramirez-Farias et al., 2008 |
|  | R | ACCTTCCTCCGTTTTGTCAAC |  |  |  |
| *R. albus* | F | CCCTAAAAGCAGTCTTAGTTCG | 176 | 100 | Koike et al., 2007 |
|  | R | CCTCCTTGCGGTTAGAAC |  |  |  |
| *R. flavefaciens* | F | CGAACGGAGATAATTTGAGTTTACTTAGG | 132 | 100 | Denman and McSweeney, 2006 |
|  | R | CGGTCTCTGTATGTTATGAGGTATTACC |  |  |  |
| *F. succinogenes* | F | GTTCGGAATTACTGGGCGTAAA | 121 | 100 | Denman and McSweeney, 2006 |
|  | R | CGCCTGCCCCTGAACTATC |  |  |  |
| *B. fibrisolvens* | F | GCCTCAGCGTCAGTAATCG | 187 | 103 | Stevenson and Weimer, 2007 |
|  | R | GGAGCGTAGGCGGTTTTAC |  |  |  |
| *frd*A^1^ | F | TGACYGARGGTTGYCG | 348 | 90 | Hattori and Matsui, 2008 |
|  | R | CRCCCATRGWRTAGTG |  |  |  |
| Fungi | F | GAGGAAGTAAAAGTCGTAACAAGGTTTC | 112 | 105 | Denman and McSweeney, 2006 |
|  | R | CAAATTCACAAAGGGTAGGATGATT |  |  |  |
| Protozoa | F | GCTTTCGWTGGTAGTGTATT | 234 | 108 | Sylvester et al., 2004 |
|  | R | CTTGCCCTCYAATCGTWCT |  |  |  |
| *mcr*A^2^ | F | TTCGGTGGATCDCARAGRGC | 160 | 104 | Denman et al., 2007 |
|  | R | GBARGTCGWAWCCGTAGAATCC |  |  |  |
| RCC^3^ | F | CAGCAGTCGCGAAAACTTC | 485 | 100 | Mihajlovski et al., 2010 |
|  | R | AACAACTTCTCTCCGGCAC |  |  |  |
| Non-RCC methanogens | F | AATTGGAKTCAACGCCGGR | 142 | 104 | DeLong, 1992 |
|  | R | TGGGTCTCGCTCGTTG |  |  |  |
| *fhs*^4^ | F | GTWTGGGCWAARGGYGGMGAAGG | 342 | 104 | Xu et al., 2009 |
|  | R | GARGAYGGWTTTGAYATYAC |  |  |  |

^1^ Fumarate reductase gene α subunit gene

^2^ Methyl CoM reductase gene α subunit gene

^3^ Rumen cluster C

^4^ Formyltetrahydrofolate synthetase gene

**Reference**

Denman, S. E., Tomkins, N. W., and McSweeney, C. S. (2007). Quantitation and diversity analysis of ruminal methanogenic populations in response to the antimethanogenic compound bromochloromethane. FEMS Microbiol. Ecol. 62, 313-322. j.1574-6941.2007.00394.x

DeLong, E. F. (1992). Archaea in coastal marine environments. P. Natl. Acad. Sci. USA. 89, 5685-5689. doi: 10.1073/pnas.89.12.5685

Denman, S. E., and McSweeney, C. S. (2006). Development of a real‐time PCR assay for monitoring anaerobic fungal and cellulolytic bacterial populations within the rumen. FEMS Microbiol. Ecol. 58, 572-582.

Hattori, K., and Matsui, H. (2008). Diversity of fumarate reducing bacteria in the bovine rumen revealed by culture dependent and independent approaches. Anaerobe 14, 87-93. doi: 10.1016/j.anaerobe.2007.12.002

Koike, S., Yabuki, H., and Kobayashi, Y. (2007). Validation and application of real‐time polymerase chain reaction assays for representative rumen bacteria. J. Anim. Sci. 78, 135-141.

Mihajlovski, A., Doré, J., Levenez, F., Alric, M., and Brugère, J. F. (2010). Molecular evaluation of the human gut methanogenic archaeal microbiota reveals an age‐associated increase of the diversity. Env. Microbiol. Rep. 2, 272-280.

Ramirez-Farias, C., Slezak, K., Fuller, Z., Duncan, A., Holtrop, G., and Louis, P. (2008). Effect of inulin on the human gut microbiota: stimulation of Bifidobacterium adolescentis and Faecalibacterium prausnitzii. Br. J. Nutr. 101, 541-550. doi: 10.1017/S0007114508019880

Stevenson, D. M., and Weimer, P. J. (2007). Dominance of Prevotella and low abundance of classical ruminal bacterial species in the bovine rumen revealed by relative quantification real-time PCR. Appl. Microbiol. Biotechnol. 75, 165-174. doi: 10.1007/s00253-006-0802-y

Sylvester, J. T., Karnati, S. K., Yu, Z. T., Morrison, M., and Firkins, J. L. (2004). Development of an assay to quantify rumen ciliate protozoal biomass in cows using real-time PCR. J. Nutr. 134, 3378-3384.

Xu, K., Liu, H., Du, G., and Chen, J. (2009). Real-time PCR assays targeting formyltetrahydrofolate synthetase gene to enumerate acetogens in natural and engineered environments. Anaerobe 15, 204-213. doi: 10.1016/j.anaerobe.2009.03.005
